# The Role of Ductal Lavage Cytology in the Diagnosis of Breast Cancer

**DOI:** 10.34172/aim.2022.118

**Published:** 2022-11-01

**Authors:** Gülçin Harman Kamalı, Sedat Kamali

**Affiliations:** ^1^Department of Pathology, University of Health Sciences Prof. Dr. Cemil Taşcıoğlu City Hospital, İstanbul, Turkey; ^2^Department of General Surgery, University of Health Sciences Prof. Dr. Cemil Taşcıoğlu City Hospital, İstanbul, Turkey

**Keywords:** Breast carcinoma, Ductal lavage cytology, Ductography, Nipple discharge

## Abstract

**Background:**

Nipple discharge is a common finding which may be a symptom of breast cancer, but it is mostly caused by benign causes. A surgical biopsy followed by a histopathological examination is considered to be the gold standard for the diagnosis of pathological nipple discharge. Non-surgical diagnostic methods should be considered to reduce the need for intervention. Ductal lavage cytology (DLC) is performed by washing and examining the ductal discharge. The usefulness of examining spontaneous discharge is controversial. This study’s aim is to evaluate the usefulness in surgical decision-making of ultrasonography (USG), mammography (MMG), magnetic resonance imaging (MRI), ductography, and DLC in patients with pathological nipple discharge.

**Methods:**

Between 2011 and 2018, we retrospectively analyzed 141 patients with pathological nipple discharge who were planned to undergo a surgical procedure and were found to have pathology. In our study, the diagnostic efficiency of DLC for breast cancer diagnosis was compared with USG, MMG, MRI, and ductography.

**Results:**

USG was performed in all patients, MMG in 51, MRI in 56, ductography in 46 patients, and cytological samples were taken from 63 patients. Twelve of 17 patients with malignant pathology were reported cytologically as suspected malignancy. The sensitivity of DLC was 70.5% (95% CI: 0.489–0.922), and its specificity was 94.1% (95% CI: 0.862–1.020).

**Conclusion:**

Numerous studies report that cytology is not adequate for final diagnosis. Negative cytology does not exclude the possibility of malignancy, and positive results do not help in the differential diagnosis.

## Introduction

 Nipple discharge is the third most common reason for visiting a physician after mastalgia and breast mass. This complaint constitutes approximately 10% of all gynecology visits.^1^ Nipple discharge usually has a benign cause. Unilateral, bloody, serous, or persistent discharge from a single duct is considered a pathological nipple discharge. In studies on women with this type of discharge, breast cancer incidence has been reported in a wide range of 0.5–21.3%.^2-5^ Surgical treatment is not required in the majority of patients.^5^ Thus, the question of which patients should be considered for surgical treatment plays an important role. There is no consensus on the various diagnostic tests and surgical procedures to confirm or exclude breast cancer in patients presenting with nipple discharge.^6^ Additional methods are needed, especially in the early stages of breast cancer. Generally, patients who undergo surgery are determined by biopsy, which is a relatively expensive, and an invasive procedure.

 Surgical biopsy followed by histopathological examination is considered the gold standard for diagnosing pathological nipple discharge.^1^ In this regard, radiological methods, nipple aspirate fluid (NAF), and examination of ductal lavage fluid in various ways are auxiliary diagnostic methods. NAF is obtained by provoking breast discharge with a massage and examined cytologically afterward. Unfortunately, the limited number of cellular elements obtained by this relatively simple method results in traditionally high false-negative rates in the cytological analysis. It limits the utility of this diagnostic method. In studies conducted based on this argumentation, more epithelial cells can be collected via cannulation and lavage of the affected duct with physiological saline as in ductal lavage cytology (DLC); it has been reported that cytological atypia can be seen in patients with high risk for breast cancer up to 24%.^7^

 In our study, the data of patients who had undergone surgery due to pathological nipple discharge based on radiological, ductoscopic, or cytological findings were evaluated retrospectively. This retrospective study’s primary purpose is to determine the usefulness of ultrasonography (USG), mammography (MMG), magnetic resonance imaging (MRI), ductography, and DLC in surgical decision-making in patients presenting with pathological nipple discharge.

## Materials and Methods

**Figure 1:**
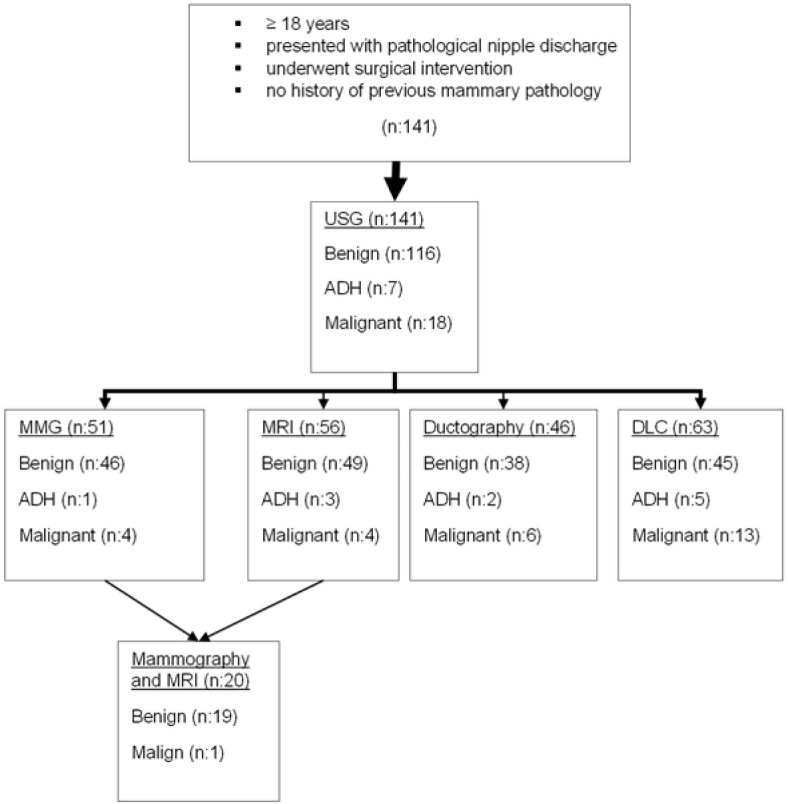
Diagnostic Tests with Surgical Pathology Results. USG, Ultrasonography; ADH, Atypical ductal hyperplasia; MMG, Mammography; MRI, Magnetic resonance imaging; DL, Ductal lavage.

 Between the years 2011 and 2018, under the ethics committee approval number 37/2021, patients who were admitted to Prof. Dr. Cemil Taşcıoğlu City Hospital, İstanbul with pathological nipple discharge were investigated retrospectively via simple random sampling. Patients who had spontaneous persistent single duct nipple discharge and underwent surgical intervention were included in this study. Subjects with known mammary pathology were excluded from this study. After collection medical history and physical examination, all patients were examined with USG. As a secondary imaging technique, patients older than 40 were advised for MMG, and patients younger than 40 years of age were advised for MRI. In case of suspicious USG findings suggestive of a papilloma, the patients underwent ductoscopy and/or DLC. Imaging data and surgical specimens along with cytological material were evaluated by an experienced blinded radiologist and pathologist, respectively (Figure 1).

 For local anesthesia, subcutaneous periareolar injections of 1% lidocaine were applied with a 30-gauge needle beforehand. Ductoscopies were performed in the supine position, and the site was disinfected after anesthesia. Specimens were collected from ductal orifices by inserting an 18 G venous cannula to a depth of 1–1.5 cm. A dilatator was used to facilitate cannulation if needed. After 2-5 mL of saline was administered, the aspirate fluid was collected into the tube. This process was repeated until about 8–10 mL of liquid was collected.

 After cytocentrifugation (Cyto-Tek 2500, 1500 RPM, 5 min), PAP (Papanicolaou) staining was used for cytological evaluation. Cytological findings were grouped about concerning ductal cells as insufficient (fewer than ten epithelial cells or technically unsuitable samples), negative (benign) and positive (observation of malignant cells or atypical cells) smear. Postoperative histopathology findings were grouped as benign, potential neoplastic and/or malignant lesions (PNMLs).

**Figure 2:**
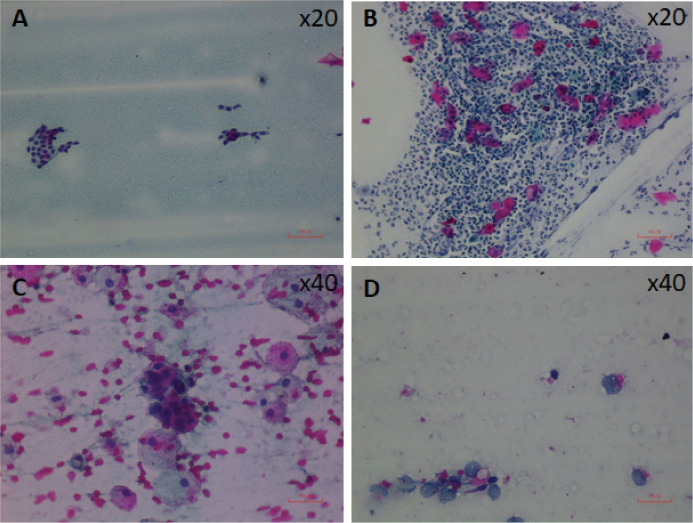
Four Lavage Cytology Samples From Different Patient Groups. **(A) ** x20 PAP, Uniform-looking monolayer benign ductal epithelial cells on a proteinaceous background ** (B) ** x20 PAP, Squamous cells with many neutrophils, fewer lymphocytes and macrophages in mastitis setting ** (C) ** x40 PAP, Atypical epithelial cells with a large core, eosinophilic cytoplasm and macrophages on a hemorrhagic background ** (D) ** x40 PAP, Isolated malignant epithelial cells with large nuclei, narrow cytoplasm showing different nuclei sizes.

 Cytology specimens were collected from 63 patients with pathological nipple discharge and evaluated according to the following criteria.^8^ Specimens containing degenerated foamy macrophages, ductal cells with apocrine metaplasia, inflammatory cells, myoepithelial cells, or epithelial cells that form tightly organized clusters were diagnosed as benign. Specimens with varying degrees of nuclear growth and hyperchromasia, irregular nuclear membrane, large and multiple nucleoli, necrotic background, and atypical cells with increased nucleocytoplasmic ratio were defined as malignant. Malignant cells were often dispersed individually without making groups and observed with patches of acute inflammatory cells, erythrocytes, and necrotic debris (Figure 2).

 The results were evaluated together with the remaining data of the patients. SPSS (Windows version 18.0; SPSS Inc, Chicago, IL, USA) was used for statistical analysis. To determine the differences between subgroups, the Mann-Whitney U, Wilcoxon rank sum and Fisher’s Chi-square tests were used for numerical and categorical data.

## Results

 Based on the diagnostic methods used, 141 patients underwent a surgical procedure. The mean age of patients was 47.7 years (Median: 46, Min: 20, Max: 75, SD: 11.6). 73 patients were perimenopausal, and 68 patients were postmenopausal. The patients’ mean body mass index (BMI) was 28.41 (Min: 16.8, Max: 47.33, SD: 4.92).

 The mean duration of discharge was 15.3 months (10 days – 180 months). Radiological (USG, MMG, MRI, ductography) examination, clinical examination and ductal lavage (before ductoscopy) were performed. In patients with a surgical indication, major duct excision was applied primarily. USG was performed in all patients, MMG in 51 (36%), MR in 56 (39.7%), ductography in 46 (32.6%) patients and cytological samples were taken from 63 (44.6%) patients. In 12 samples taken from these 63 patients, not enough cells were found for cytology. Surgical histopathology was benign in 116 (82.3%). Atypical ductal hyperplasia (ADH) was found in seven (5%) and malignancy was detected (nine invasive carcinomas, nine ductal carcinomas in situ) in 18 (12.8%) patients (Table 1).

**Table 1:** Final Pathology Results Based on Preoperative Evaluation

**Test**	**Benign** **Histopathology**	**Atypical** **Histopathology**	**Malignant** **Histopathology**
USG (n = 141)	116 (82.2%)	7 (5%)	18 (12.8%)
MMG (n = 51)	46 (90.2%)	1 (2%)	4 (7.8%)
MRI (n = 56)	49 (87.5%)	3 (5.4%)	4 (7.1%)
Ductography (n = 46)	38 (82.6%)	2 (4.3%)	6 (13%)
Ductal cytology (n = 63)	45 (71.4%)	5 (7.9 %)	13 (20.6 %)

USG, Ultrasonography; MMG, Mammography; MRI, Magnetic resonance imaging.

 When the patient groups were compared in terms of benign and PNMLs, no difference was observed between the groups regarding age, menopausal status, duration of discharge, the color of discharge, and BMI.

 Approximately 18% of patients were found to have breast carcinoma.

 Based on USG findings such as irregular shape or architectural distortion, six patients (4.3%) were suspected of malignancy. The sensitivity of USG to detect PNMLs pathology was 20% (95% CI: 0.043–0.356) and the specificity of USG was 99% (95% CI: 0.974–1.008). USG had a positive predictive value (PPV) of 83% and a negative predictive value (NPV) of 85% (Table 2).

**Table 2:** Diagnostic Indices Based on Preoperative Study

	**Sensitivity (95% CI)**	**Specificity (95% CI)**	**PPV**	**NPV**	**TN**	**TP**	**FN**	**FP**	**LR+**	**LR-**
USG	0.200 (0.043- 0.356)	0.990 (0.974- 1.008)	0.833	0.851	115	5	20	1	23.2	0.8
MMG	0.400 (0.029 - 0.829)	0.695 (0.562 -0.826)	0.125	0.914	32	2	3	14	1.314	0.862
USG and MMG	0.600 (0.170 - 1.029)	0.695 (0.562 -0.828)	0.176	0.941	32	3	2	14	1.971	0.575
Ductography	0.375 (0.039 - 0.710)	0.868 (0.760 -0.975)	0.375	0.868	33	3	5	5	2.85	0.719
MRI	0.428 (0.061 - 0.795)	0.795 (0.683 -0.908)	0.23	0.906	39	3	4	10	2.1	0.717
DLC	0.705 (0.489 - 0.922)	0.941 (0.862 -1.020)	0.857	0.864	39	12	5	2	12	0.312
USG and MRI	0.571 (0.204 -0.938)	0.795 (0.683 -0.908)	0.285	0.928	39	4	3	10	2.8	0.538
MMG, USG and MRI	1 (1 – 1)	0.421 (0.199 -0.643)	0.083	1.000	8	1	0	11	1.727	0

95% CI, Confidence Interval 95; PPV, Positive Predictive Value; NPV, Negative predictive value; TN, True negative; TP, True positive; FN, False negative, FP: False positive, LR(-): Likelihood ratio (-), LR(+): Likelihood ratio (+), MMG: mammography USG: Ultrasonography, MRI: Magnetic resonance imaging; DLC, ductal lavage cytology.

 Fifty-one patients had preoperative USG and MMG. Thirty-four (66.7%) of the 51 subjects had benign mammographic findings, while 17 (33.3%) subjects showed suspicious findings of malignancy. Among those 51 subjects, 46 (90.2%) had benign pathological findings, whereas five (10%) showed pathological findings suggestive of PNML. Sole MMG sensitivity for detecting malignant ductal pathology was 40% (95% CI: 0.029–0.829), and the specificity was 69.5% (95% CI: 0.562–0.826). For this diagnostic method, PPV was 16.7%, and NPV was 95.1%. The sensitivity of MMG and USG, when combined for diagnosis of ductal PNMLs, was 60% (95% CI: 0.562–0.826) with a specificity of 69.5%, and PPV was calculated as 17.6%, while NPV was 94.1% (Table 2).

 Fifty-six patients underwent USG and MRI preoperatively. Benign MRI findings were reported in 43 patients (76.8%), while suspicious findings were reported in 13 patients (23.2%). The pathological examination revealed benign findings in 49 subjects (87.5%) and pathological findings suggestive of PNML in seven subjects (12.5%). The sensitivity of MRI for detecting PNMLs ductal pathology was 42.8% (95% CI: 0.061–0.795), and the specificity was 79.5% (95% CI: 0.683–0.908), with a PPV of 23%, and NPV of 90.6%. The combined sensitivity of USG and MRI for detecting ductal pathology was 57.1%, and the specificity was 79.5%. For this diagnostic method, PPV was 28.5%, and NPV was 92.8% (Table 2).

 Twenty patients underwent USG, MRI and MMG preoperatively. Nineteen (95%) subjects had benign radiological findings, while one (5%) subject showed suspicious findings of PNMLs. Among 20 tests, eight (40%) had benign, and 12 (60%) showed PNMLs surgical pathology. The sensitivity of test combinations for finding PNMLs ductal pathology was 100% (95% CI: 1–1), its specificity was 42.1% (95% CI: 0.199–0.643) and PPV was calculated as 8.3% and NPV as 100%.

 Preoperative ductography was performed on 46 subjects. Eight subjects (17.3%) showed suspicious findings of malignancy. Among 46 ductographies, 38 (82.6%) had benign findings, whereas eight (17.4%) showed findings in favor of PNMLs. The sensitivity of ductography for diagnosis of malignant ductal pathology was 37.5% (95% CI: 0.039–0.710), and its specificity was 86.8% (95% CI: 0.760–0.975) while PPV reached 37.5% and NPV reached 86.8% (Table 2).

 Cytology specimens were evaluated according to the above-mentioned criteria. In 12 (19%) patients, no ductal cells were observed, although malignant surgical pathology was reported in one patient (8.3%). Of 37 (58.7%) patients who had initially reported in favor of benign cytology, 32 (86.5%) were described in the final pathology report as benign (11 solitary papillomas, eight multiple papillomas, nine mastitis, three ductal hyperplasias, one ductal ectasia), and five (13.5%) were described as PNMLs (two ductal carcinomas in situ, one invasive carcinoma, two ADHs). From 14 (22.2%) cytology specimens described or suspected as malignant, 12 (85.7%) were reported in the surgical pathology as PNMLs two (14.3%) as benign (Table 3). The sensitivity of cytological examination for detection of PNMLs was 70.5% (95% CI: 0.489–0.922) while specificity was 94.1% (95% CI: 0.0862–1.020). PPV was calculated as 85.7% and NPV as 86.4% (Table 2).

**Table 3:** Surgical pathology * Cytology: Cross tabulation

			**Cytology all**			
			**Malign or Suspect**	**No Ductal Cell**	**Benign**	**Total**
Surgical pathology	Solitary papilloma	Count	2	4	11	**17**
		% Cytology	14.3%	33.3%	29.7%	27.0%
	Multiple papilloma	Count	0	2	8	**10**
		% Cytology	0.0%	16.7%	21.6%	15.9%
	Mastitis	Count	0	3	9	**12**
		% Cytology	0.0%	25.0%	24.3%	19.0%
	Ductal carcinoma in situ	Count	5	0	2	**7**
		% Cytology	35.7%	0.0%	5.4%	11.1%
	Invasive Carcinoma	Count	4	1	1	**6**
		% Cytology	28.6%	8.3%	2.7%	9.5%
	Atypical ductal hyperplasia	Count	3	0	2	**5**
		% Cytology	21.4%	0.0%	5.4%	7.9%
	Ductal hyperplasia	Count	0	1	3	**4**
		% Cytology	0.0%	8.3%	8.1%	6.3%
	Ductal ectasia	Count	0	1	1	**2**
		%Cytology	0.0%	8.3%	2.7%	3.2%
**Total**		Count	**14**	**12**	**37**	**63**
		% Cytology	100.0%	100.0%	100.0%	100.0%

## Discussion

 Malignant or benign lesions can cause nipple discharge. Although there is mostly a non-malignant causality behind it, it is still a concerning issue for patients and physicians. Firstly, less invasive examinations such as USG, MMG, MRI, ductography and cytological examination are utilized. None of those findings alone may be sufficient, but more than one examination may still not deliver final results. A standard diagnosis algorithm for patients with pathological nipple discharge is yet to be defined.^9,10^

 In this study, 12.8% of DLC findings were malignant, and 5% were atypical in the final pathology report, which supports that the discharge cause is usually (82.2%) a benign disease (papilloma, ductal ectasia). The carcinoma rate in this study was consistent with the literature reporting the carcinoma incidence between 9.3-21.3% in patients with nipple discharge.^2–6^

 The sensitivity of USG in detecting malignant pathology was found to be very low (20%), which corroborates that USG is preferably complementary to MMG in breast cancer diagnosis.^11^ Our study shows that MMG alone, with low PPV (12.5%), is not a reliable method in determining the cause of nipple discharge, as mentioned in other studies, MMG with 40% sensitivity in detecting malignant ductal pathology, is a limited screening test to evaluate nipple discharge.^1^

 Although MRI is a valuable diagnostic tool, it is not among routine examinations. Bahl et al discussed the value of MRI in women with nipple discharge.^12^ While Patel advised MRI in patients whose USG and MMG did not yield any results, Berger stated that it should be conducted prior to ductography.^13,14^ While MRI is not a routine examination; it has been more suitable for patients at younger ages than MMG. When MRI findings were compared with pathology results, it was seen that MRI had a relatively low specificity (79.5%) as stated in other studies (72%),^15^ which allows the argument that sole MRI with a sensitivity of 50% and NPV of 16.5% is at least as useful a modality as other radiological imaging methods.

 Although ductography is not one of the primary diagnostic methods, it is a valuable examination in patients with nipple discharge. In some reports, the sensitivity of ductography is 93%, and its specificity is 39%.^5^ In our study, the sensitivity of ductography was found to be as low as 37.5%, but the specificity was found to be as high as 86.8%. While ductography was performed on 200 patients in the study with higher sensitivity, we performed this technique on 46 patients which may explain our findings. According to our findings, ductography is more effective in differentiating the non-malignant cause of discharge after excluding any malignancies. However, a PPV of 37.5% still shows that it is not sufficient as a first-line single diagnostic tool for malignant ductal pathology.

 The criteria for nipple discharge cytology are well defined.^8^ Benign smears are generally lower in cell numbers, and non-epithelial cells comprise approximately 50% of the ductal lavage fluid cells. Unless there are multiple atypical cells, malignant cytology should not be considered.^16^ However, the benign cytology of the discharge does not exclude malignancy. Keeping in mind that the cytological evaluations’ false-negative rates reach up to 50%,^17,18^ mostly caused by adenocarcinoma associated with mastitis, positive cytology with malignant cells is still beneficial for the further therapeutic.

 Although microscopy has been used to evaluate NAF for the presence of blood, the relationship between bloody discharge and malignancy is not clear. Erythrocytes can be traced in both benign and malignant samples. While some clinicians reported higher malignancy rates in patients with bloody nipple discharge,^16,17^ others did not find this relationship.^6,19-21^ In our study, no difference was found between malignant and benign groups regarding discharge color.

 There are two methods for obtaining nipple discharge specimens. For NAF, the specimen is collected via all of the mammary ducts with the aid of massage, while for DLC, it is obtained by washing specifically the ductal canal with pathological discharge. In both methods sensitivity has a wide margin range (26.7–85%).^1^ In addition to studies reporting the sensitivity and specificity of cytology as 46% and 95%^22^ in patients with nipple discharge, there are other retrospective studies reporting sensitivity as low as 11% and specificity as 76%,^1^ and therefore claiming that the evaluation of nipple discharge is not sufficient. The diagnostic values of cytological examination were higher in our study (sensitivity: 70.5%, specificity: 94.1%, PPV: 85.7%, NPV: 86.4%) compared to previous studies. Moreover, none of our patients yielded false-positive DLC results, most probably due to selective cannulation of the affected duct.

 Having the same senior pathologist perform all cytological evaluations could reduce interobserver variability and explain our higher sensitivity and specificity. Another explanation for previous studies lower lower sensitivity and specificity may be the different cohort characteristics. Our analysis also included not all patients with nipple discharge, but only patients with pathological nipple discharge, which was confirmed by surgical biopsy. The specificity of cytology in our study may be increased, since our definition of bloody discharge was based on cytology rather than a basic hemoccult test.

 One of the limitations of this study was retrospective data collection, and the possible inconsistency or incomplete recording of clinical findings. Second, the small sample size limited the power of our study. Finally, smaller number of sufficient cytology samples limited the significance analysis. Since the sample taken for cytology is usually in very small, its sensitivity may also be impaired.

 In conclusion,numerous studies have demonstrated the inability of cytology to predict eventual surgical pathology.^6,7^ The use of cytology for diagnosis is always limited since a negative result cannot rule out malignancy and a positive result for malignancy cannot distinguish between in situ and invasive ductal carcinoma.^9^ Evaluation of patients with nipple discharge by multiple diagnostic tools increases the rate of correct diagnosis. Before performing a surgical biopsy on a patient with abnormal nipple discharge, the results of MMG, MRI, ductography, and DLC should be evaluated together, and the decision for surgery as a final treatment should be taken in light of these findings.
